# Supplementary material for: COVID-19 Immunisation, Willingness to Be Vaccinated and Vaccination Strategies to Improve Vaccine Uptake in Australia
Source: Vaccines (Basel). 2021 Dec 11;9(12):1467. doi: 10.3390/vaccines9121467 (PMC8704895; doi:10.3390/vaccines9121467)
Supplement: Supplementary file 1 [file vaccines-09-01467-s001.zip › vaccines-1458846-supplementary.pdf]

Supplementary data

Socio-Economic Index For Areas (SEIFA) quintiles were presented according to the definition from the Australian Bureau of Statistics using the respondent's postcode and suburb. The respondent's country of birth/Culturally and Linguistically Diversity was divided into two broad categories: English speaking countries and non-English speaking countries. Respondents who were born in Australia, the United Kingdom, Ireland, the United States of America, New Zealand, Canada, and South Africa are included in the English-speaking background category. All other respondents were included in the non-English speaking background category. Health outcomes presented in the analyses were self-reported according to whether the respondent had even been told by a doctor or nurse they had a chronic medical condition. Aboriginal was used in this document respectfully as an all-encompassing term for Aboriginal and Torres Strait Islander people, health, and culture.

Table S1: Survey questions

Questions	Responses
**The next few questions are about the COVID-19 vaccine. Since March, South Australia has been rolling out the vaccine starting with those who are most vulnerable to the effects of COVID-19 or who have the highest risk of being exposed to, or spreading the virus.**	
**1. Which of the following statements about the COVID-19 vaccine best apply to you?**<br>**(Single response)**	1. I will be getting vaccinated when it becomes available to me<br>2. I will not be getting vaccinated it becomes available to me<br>3. I am undecided whether or not I will be getting vaccinated when it becomes available to me<br>4. I have been vaccinated (one or two doses)<br>5. Prefer not to say
**2. Do you think COVID-19 vaccination or proof of vaccination should be required for:**<br>**(Select all that apply)**	1. International travelling<br>2. Domestic travelling<br>3. Should not be required<br>4. Don't know<br>5. Prefer not to say
**3. If you are a parent or caregiver of a child/children aged less than 16 years, please answer this question. If the COVID-19 vaccine is safe, effective and approved to use in children by the government, how likely would you be to get your child/children vaccinated?**<br>**(Single response)**	1. Very likely<br>2. Somewhat likely<br>3. Not very likely<br>4. Not at all likely<br>5. Don't know<br>6. Prefer not to say<br>7. Not a parent or care-giver of a child <16 years
**How much do you agree or disagree with the following statements:**	
**4. COVID-19 vaccination should be made mandatory by the Government.**	1. Strongly Agree<br>2. Somewhat agree<br>3. Neither agree or disagree<br>4. Somewhat disagree<br>5. Strongly disagree<br>6. Don't know<br>7. Prefer not to say
**5. Tailored vaccine reminder message/letter should be sent to everyone.**<br>**(Single response)**	1. Strongly Agree<br>2. Somewhat agree<br>3. Neither agree or disagree<br>4. Somewhat disagree<br>5. Strongly disagree

	6. Don't know<br>7. Prefer not to say
**6. COVID-19 vaccination or proof of vaccination should be required for visiting Residential Aged Care Homes and working in a hospital or healthcare clinic.**<br>**(Single response)**	1. Strongly Agree<br>2. Somewhat agree<br>3. Neither agree or disagree<br>4. Somewhat disagree<br>5. Strongly disagree<br>6. Don't know<br>7. Prefer not to say

Table S2: Weighted and adjusted odds ratios of parental willingness to get their child/children vaccinated

**Parental vaccine willingness**	**aOdds Ratio**	**95%CI**
Age	1.00	0.99-1.02
**SEIFA**		
lowest quintile	Ref	
low quintile	0.85	0.47-1.53
middle quintile	0.71	0.40-1.28
high quintile	0.86	0.47-1.58
highest quintile	1.05	0.57-1.96
**Education level**		
Lower than Year 12 education	Ref	
≥Y12/TAFE/certificate/diploma	0.91	0.51-1.62
Degree or higher	0.87	0.48-1.58
**Chronic medical conditions**		
No	Ref	
Yes	1.15	0.78-1.70
**Willingness of taking vaccination themselves**		
Will not get vaccinated/undecided	Ref	
Will get vaccinated/have already been vaccinated	14.10	9.15-21.71

Table S3: Weighted and adjusted odds ratios of agreeing that COVID vaccination should be required for international travelling

**International travelling**	**aOdds Ratio**	**95%CI**
**Age group**		
18-29yrs	Ref	
30-49yrs	0.83	0.58-1.19
50-69yrs	1.16	0.79-1.70
≥70yrs	2.47	1.48-4.12
**Gender**		
Male	Ref	
Female	1.25	0.97-1.60
**SEIFA**		
lowest quintile	Ref	

International travelling	aOdds Ratio	95%CI
low quintile	1.23	0.84-1.78
middle quintile	1.70	1.16-2.48
high quintile	1.31	0.89-1.93
highest quintile	1.74	1.15-2.61
**Marital status**		
Married/Living with partner	Ref	
Single	0.78	0.60-1.02
**Employment**		
Unemployed	Ref	
Employed	1.43	0.92-2.22
Others	1.46	0.90-2.37
**Area of residence**		
Metro. Adelaide	Ref	
SA Country	1.30	0.96-1.76
**Chronic medical conditions**		
No	Ref	
Yes	1.09	0.83-1.44

Table S4: Weighted and adjusted odds ratios of agreeing that COVID vaccination should be required for domestic travelling

Domestic travelling	aOdds Ratio	95%CI
**Age group**		
18-29yrs	Ref	
30-49yrs	0.98	0.76-1.27
50-69yrs	1.53	1.18-1.99
≥70yrs	2.37	1.69-3.32
**Gender**		
Male	Ref	
Female	1.02	0.86-1.22
**Education level**		
Lower than Year 12 education	Ref	
≥Y12/TAFE/certificate/diploma	1.05	0.83-1.32
Degree or higher	1.23	0.96-1.59
**Employment**		
Unemployed	Ref	
Employed	0.75	0.52-1.09
Others	1.07	0.72-1.61
**Chronic medical conditions**		
No	Ref	
Yes	1.25	1.03-1.52

Table S5: Adjusted odds ratios of strongly agreeing that COVID-19 vaccination should be made mandatory by the Government

Mandatory vaccination	aOdds Ratio	95%CI
**Age**		
18-29yrs	Ref	
30-49yrs	1.18	0.86-1.60
50-69yrs	1.44	1.06-1.97
≥70yrs	2.48	1.72-3.55
**Gender**		
Male	Ref	
Female	0.73	0.61-0.88
**Aboriginal status**		
No		
Yes		
**SEIFA**		
lowest quintile	Ref	
low quintile	0.72	0.53-0.96
middle quintile	0.88	0.65-1.19
high quintile	0.75	0.55-1.03
highest quintile	0.82	0.60-1.13
**Marital status**		
Married/de facto		
Single		
**Education level**		
Lower than Year 12 education	Ref	
≥Year 12/ TAFE/ certificate/ diploma	0.98	0.78-1.23
Degree or higher	0.83	0.64-1.08
**Employment**		
Unemployed	Ref	
Employed^	0.68	0.46-1.01
Others^^	1.12	0.73-1.71
**COB (Country of Birth)**		
COB English main language	Ref	
COB Non English speaking	1.36	1.04-1.77
**Area of residence**		
Metro. Adelaide	Ref	
SA Country	1.14	0.92-1.42
**Chronic medical conditions**		
No	Ref	
Yes	0.89	0.72-1.10

Table S6: Adjusted odds ratios of strongly agreeing that tailored vaccine reminders should be sent to everyone

Tailored vaccine reminders	aOdds Ratio	95%CI
**Age group**		
18-29yrs	Ref	
30-49yrs	1.34	1.02-1.76

Tailored vaccine reminders	aOdds Ratio	95%CI
50-69yrs	1.17	0.90-1.53
≥70yrs	1.34	0.98-1.84
**Marital status**		
Married/Living with partner	Ref	
Single	0.89	0.75-1.07
**Education level**		
Lower than Year 12 education	Ref	
≥Y12/TAFE/certificate/diploma	1.05	0.85-1.30
Degree or higher	1.20	0.95-1.51
**Employment**		
Unemployed	Ref	
Employed	0.87	0.61-1.26
Others	1.15	0.78-1.69
**COB (Country of Birth)**		
COB English main language	Ref	
COB Non English speaking	1.24	0.98-1.58

Table S7: Adjusted odds ratios of strongly agreeing that COVID-19 vaccination should be required for visiting Residential Aged Care Homes and working in healthcare settings

Vaccination requirements for visiting Residential Aged Care Homes and working in healthcare settings	aOdds Ratio	95%CI
**Age group**		
18-29yrs	Ref	
30-49yrs	0.98	0.74-1.30
50-69yrs	1.10	0.83-1.46
≥70yrs	1.74	1.22-2.49
**Aboriginal status**		
No	Ref	
Yes	0.68	0.35-1.30
**SEIFA**		
lowest quintile	Ref	
low quintile	0.96	0.71-1.30
middle quintile	1.06	0.78-1.45
high quintile	0.90	0.65-1.23
highest quintile	1.40	1.02-1.92
**Marital status**	Ref	
Married/Living with partner		
Single	0.85	0.70-1.04
**Education level**		
Lower than Year 12 education	Ref	
≥Y12/TAFE/certificate/diploma	0.86	0.67-1.10
Degree or higher	1.00	0.76-1.33
**Employment**		
Unemployed	Ref	

**Vaccination requirements for visiting Residential Aged Care Homes and working in healthcare settings**	**aOdds Ratio**	**95%CI**
Employed	0.98	0.66-1.44
Others	1.48	0.98-2.26
